# Galanin inhibits GLP‐1 and GIP secretion via the GAL_1_ receptor in enteroendocrine L and K cells

**DOI:** 10.1111/bph.13407

**Published:** 2016-02-08

**Authors:** Arianna Psichas, Leslie L Glass, Stephen J Sharp, Frank Reimann, Fiona M Gribble

**Affiliations:** ^1^ Metabolic Research Laboratories and MRC Metabolic Diseases Unit, WT‐MRC Institute of Metabolic Science, Addenbrooke's HospitalUniversity of CambridgeCambridgeCB2 0QQUK; ^2^ MRC Epidemiology Unit, WT‐MRC Institute of Metabolic Science, Addenbrooke's HospitalUniversity of CambridgeCambridgeCB2 0QQUK

## Abstract

**Background and Purpose:**

Galanin is a widely expressed neuropeptide, which in the gut is thought to modulate gastrointestinal motility and secretion. We aimed to elucidate the poorly characterised mechanisms underlying the inhibitory effect of galanin and the potential involvement of G‐protein coupled inwardly rectifying potassium, K_ir_3, (GIRK) channels in glucagon‐like peptide 1 (GLP‐1) and glucose‐dependent insulinotropic polypeptide (GIP) secretion.

**Experimental Approach:**

Purified murine L and K cells were analysed for expression of galanin receptors and GIRK subunits. Hormone secretion was measured from primary murine intestinal cultures. Intracellular cAMP was monitored in primary L cells derived from mice expressing the *Epac2camps* sensor under the control of the proglucagon promoter.

**Key Results:**

Galanin receptor 1 (GAL_1_, *Galr1*) and GIRK channel 1 (K_ir_3.1, *Kcnj3*) and 4 (K_ir_3.4, *Kcnj5*) mRNA expression was highly enriched in K and L cells*.* Galanin and a selective GAL_1_ receptor agonist (M617) potently inhibited GLP‐1 and GIP secretion from primary small intestinal cultures. In L cells, galanin significantly inhibited the forskolin‐induced cAMP response. The GIRK1/4 activator ML297 significantly reduced glucose‐stimulated and IBMX‐stimulated GLP‐1 secretion but had no effect on GIP. The GIRK blocker tertiapin‐Q did not impair galanin‐mediated GLP‐1 inhibition.

**Conclusions and Implications:**

Galanin, acting via the GAL_1_ receptor and G_i_‐coupled signalling in L and K cells, is a potent inhibitor of GLP‐1 and GIP secretion. Although GIRK1/4 channels are expressed in these cells, their activation does not appear to play a major role in galanin‐mediated inhibition of incretin secretion.

**Abbreviations**
CFP: cyan fluorescent protein
FRET: Förster resonance energy transfer
GIP: glucose‐dependent insulinotropic polypeptide (gastric inhibitory polypeptide)
GIRK: G‐protein coupled inwardly –rectifying potassium channel, K_ir_3.x
GLP‐1: glucagon‐like peptide 1
TPN‐Q: tertiapin‐Q
YFP: yellow fluorescent protein

## Tables of Links

**Table:** 

**TARGETS**
GPCRs^a^
GAL1 receptor
Voltage‐gated ion channels^b^
GIRK1/K_ir_3.1 channels
GIRK4/K_ir_3.4 channels

**LIGANDS**	
Galanin	M617
GIP, gastric inhibitory polypeptide	ML297
GLP‐1	Tolbutamide
IBMX	TPN‐Q, tertiapin‐Q

These Tables list key protein targets and ligands in this article which are hyperlinked to corresponding entries in http://www.guidetopharmacology.org, the common portal for data from the IUPHAR/BPS Guide to PHARMACOLOGY (Pawson *et al.*, 2014) and are permanently archived in the Concise Guide to PHARMACOLOGY 2015/16 (^*a,b*^Alexander *et al.*, 2015a, 2015b).

## Introduction

Galanin is a 29–30‐amino acid neuropeptide, synthesised in both the central and peripheral nervous systems including the enteric nervous system (Tatemoto *et al.*, 1983). Galanin has been shown to regulate a wide variety of physiological and pathophysiological processes (reviewed by Lang *et al.*, 2015). Within the intestine, galanin mRNA is most abundant in the duodenum with lower levels reported in the stomach, distal small intestine and colon (Kaplan *et al.*, 1988). The upper small intestine harbours enteroendocrine L and K cells responsible for secreting the gut hormones glucagon‐like peptide 1 (GLP‐1) and glucose‐dependent insulinotropic polypeptide (GIP), respectively, which have established roles in gastrointestinal function, glucose homeostasis and satiety (Campbell *et al.*, 2013). Gut peptide secretion is known to be influenced by a number of factors including nutrients, bile acids, microbial products, cytokines, hormones and gut neuropeptides (Psichas *et al.*, 2015). However, our understanding of the neuro‐hormonal regulation of GLP‐1 and GIP secretion and the interaction between the enteroendocrine system and the enteric nervous system remains limited.

There is accumulating evidence to suggest that galanin can modulate gastrointestinal motility and secretion (Lang *et al.*, 2007), although its physiological functions remain poorly understood. Galanin was demonstrated to inhibit GLP‐1 secretion from isolated vascularly perfused rat ileum (Herrmann‐Rinke *et al.*, 1996) and cholecystokinin secretion from the intestinal Secretin Tumour Cell line (STC‐1) (Chang *et al.*, 1995). However, the mechanisms underlying galanin‐mediated inhibition of gut hormone release are unclear.

Galanin has been identified as a ligand for three GPCRs so far, namely, galanin receptors 1–3 (GAL_1‐3_) (Habert‐Ortoli *et al.*, 1994; Howard *et al.*, 1997; Wang *et al.*, 1997a). These receptors are widely expressed in the nervous system, pancreas and intestine. Intriguingly, galanin receptors may also form heteromers with other GPCRs, potentially including 5‐HT receptors, neuropeptide Y receptors, α_2_‐adrenoceptors (Fuxe *et al.*, 2012; Fuxe *et al.*, 2008) and dopamine D_1_‐like receptors (Moreno *et al.*, 2011). The GAL_1_ receptor has been demonstrated to couple to the pertussis toxin‐sensitive G_i_ pathway, resulting in the inhibition of AC (Habert‐Ortoli *et al.*, 1994; Parker *et al.*, 1995; Wang *et al.*, 1997b). GAL_1_ receptor activation has also been associated with the opening of G‐protein coupled inwardly rectifying potassium, K_ir_3, (GIRK) channels in *Xenopus* oocytes (Smith *et al.*, 1998) and the inhibition of voltage‐gated Ca^2^^+^ channels in rat cultured myenteric neurons (Anselmi *et al.*, 2009). Mammals express four GIRK channel subunits: GIRK1 (K_ir_3.1), GIRK2 (K_ir_3.2), GIRK3 (K_ir_3.3) and GIRK 4 (K_ir_3.4), encoded by the genes *Kcnj3*, *Kcnj6*, *Kcnj9* and *Kcnj5* respectively (Lüscher & Slesinger, 2010). The opening of these channels renders them permeable to K^+^ ions, which would have a hyperpolarising effect on the cell. The GAL_2_ receptor is thought to be capable of interacting with both G_i_ and G_q_ proteins; activation of the latter would trigger PLC activity and inositol 1,4,5‐triphosphate formation, mediating the release of Ca^2^^+^ from intracellular stores. While GAL_3_ receptor signalling is largely uncharacterised, there is some evidence to suggest that it involves the G_i_ pathway and opening of GIRK (K_ir_3) channels (Smith *et al.*, 1998).

The identity of the galanin receptor and the intracellular signalling pathways responsible for galanin‐mediated inhibition of gut hormone secretion are currently unknown. Therefore, this study aimed to (i) characterise the expression profiles of galanin receptors and GIRK (K_ir_3) channel subunits in purified enteroendocrine L and K cells, (ii) determine the mechanism underlying galanin‐mediated inhibition of incretin release and (iii) investigate the role of GIRK (K_ir_3) channels in GLP‐1 and GIP secretion. We demonstrated that galanin, acting via the GAL_1_ receptor and G_i_‐coupled signalling in L and K cells, is a potent inhibitor of both GLP‐1 and GIP secretion. We found no evidence to suggest a role for GIRK1/4 (K_ir_3.1/3.4) channels in galanin‐mediated inhibition of gut hormone secretion.

## Methods

### Animal welfare and ethical statements

All animal care and experimental procedures conformed to the Animals (Scientific Procedures) Act 1986 Amendment Regulations (SI 2012/3039) and were approved by the local ethical review committee and. The work was performed under the UK Home Office Project Licence 70/7824. This paper complies with the ARRIVE guidelines (Kilkenny et al., 2010: McGrath and Lilley, 2015) where relevant. The experiments presented did not involve regulated procedures or the use of live animals. The mice were killed by cervical dislocation, an approved schedule 1 method. Intestinal tissue used in the experiments was obtained from mice on a C57BL6 background, which were housed in individually ventilated cages with ad libitum access to water and chow. Intestinal tissue from both male and female mice was used. This paper complies with the ARRIVE guidelines where relevant. The experiments presented did not involve regulated procedures or the use of live animals. Intestinal tissue used in the experiments was obtained from mice on a C57BL6 background, which were housed in individually ventilated cages with *ad libitum* access to water and chow. The mice were culled by cervical dislocation, an approved schedule 1 method. Intestinal tissue from both male and female mice was used.

### Creation of proglucagon promoter‐driven *Epac2camps* expressing transgenic mice

To express yellow fluorescent protein (YFP)/cyan fluorescent protein (CFP)‐based cAMP‐Förster resonance energy transfer (FRET)‐sensor *Epac2camps* (Nikolaev *et al.*, 2004), a kind gift by Martin Lohse (Institute of Pharmacology and Toxicology, University of Würzburg, Germany), under the control of the proglucagon promoter, we replaced the sequence between the proglucagon start codon in exon 2 and stop codon in exon 6 in the murine‐based BAC RP23‐343C17 (Children's Hospital Oakland Research Institute, Oakland, CA, USA) by the *Epac2camps* sequence using Red/ET recombination technology (Genebridges, Heidelberg, Germany) (Figure 3A). We were unable to simply introduce proglucagon gene specific 3′ and 5′ sequences through PCR amplification of *Epac2camps* with the primers mGLP002 and mGLP006, presumably because of duplication of the 5′ YFP sequence and the 3′ CFP sequence within the FRET sensor. Using a combination of PCR amplification and fragment subcloning of in‐house plasmids containing 5′ and 3′ proglucagon sequence around a YFP‐variant (Venus) insert (Reimann *et al.*, 2008), a construct containing 364 bp 5′ to the gcg start codon and 663 bp 3′ to the gcg stop codon in which the gcg coding sequence is replaced by the *Epac2camps* sequence was created and amplified with the primer pair FRGLU008/mGLP005 (see oligonucleotides tabulated below). Homologous recombination was achieved upon co‐transforming, an rpsLneo‐modified BAC (Reimann *et al.*, 2008) containing *Escherichia coli* DH10B clone with this PCR product and the plasmid pSC101‐BAD‐gbaA, which provides the recombination enzymes (GeneBridges). Positive recombinants were isolated using appropriate antibiotic selection and characterised by PCR and restriction analysis. Identity and correct positioning of the introduced *Epac2camps* sequence was confirmed by direct sequencing. BAC‐DNA for microinjection was purified using the large‐construct Maxi‐Prep kit (Qiagen, Manchester, UK) and dissolved at ~1–2 ng·μL^−1^ in injection buffer containing (mmol·l^−1^): 10 Tris–HCl pH 7.5, 0.1 EDTA, 100 NaCl, 0.03 spermine and 0.07 spermidine. Pronuclear injection into ova derived from C57BL6/CBA F1 parents and reimplantation of embryos into pseudopregnant females was performed by the Central Biomedical Services at Cambridge University. The DNA of pups was isolated from ear clips by proteinase K digestion and screened for the transgene by PCR using the following primer pairs: mGLP013/Epac_in1, GFP002/003 (and RM41/42, which amplifies the β‐catenin sequence used as a DNA quality control). We initially created 12 founders, of which 10 passed on the transgene. Two lines GLU‐Epac20 and GLU‐Epac21 were selected based on the brightness of intestinal L cells expressing the sensor and the observable responses of *Epac2camps*‐positive L cells to a test stimulus [forskolin (Fsk)/IBMX 10 μM each or taurolithocholate 10 μM] and were backcrossed onto C57BL6 for at least seven generations. Correct expression of *Epac2camps* in intestinal L cells was confirmed by immunohistochemistry in the GLU‐Epac20 and GLU‐Epac21 mouse lines (Figure 3B (upper small intestine), Supporting Information Table2 and Supporting Information Figure 1 (colon)). We observed that, on average, >80% of all proglucagon expressing cells also express the sensor, and >90% of all *Epac2camps*‐expressing cells also express proglucagon, without obvious differences in the two lines and in line with previous findings in GLU‐Venus mice carrying a similar transgene (Habib *et al.*, 2012).

### Expression analysis

L and K cells were isolated by FACS from GLU‐Venus (Reimann *et al.*, 2008) and GIP‐Venus (Parker *et al.*, 2009) transgenic mice, respectively, as previously described. Briefly, the small intestine (upper or lower 10 cm) or colon was digested as described in the succeeding texts for primary cultures, but using 1 mg mL^−1^ in HBSS (Sigma, Poole, UK), to obtain single cells. Cell suspensions were separated using a MoFlo Cytomation sorter (Beckman Coulter, High Wycombe, UK) (488 nm excitation) to obtain populations of Venus‐positive or control Venus‐negative cells, which were collected directly into lysis buffer for mRNA extraction. Purified K and L cell populations were over 95% pure as assessed by fluorescence microscopy and were enriched more than 2000‐fold for GIP and GLP‐1 concentrations, respectively, compared with non‐fluorescent cells, as assessed by ELISA (Parker *et al.*, 2009; Reimann *et al.*, 2008).

RNA was extracted using a microscale RNA isolation kit (Applied Biosystems, Warrington, UK) as previously described (Parker *et al.*, 2009; Reimann *et al.*, 2008). For hybridisation to mouse 430 2.0 arrays GeneChips (Affymetrix, High Wycombe, UK), RNA underwent two rounds of amplification based on *in vitro* translation from a T7 promoter induced during oligo dT‐priming (two‐cycles cDNA synthesis Kit, Affymetrix; MEGAscript T7 kit, Ambion, Austin, TX, USA), and expression levels of each probe were determined by robust multichip average analysis.

### Quantitative RT‐PCR

Extraction of RNA from purified intestinal cells was performed using an RNeasy Micro Kit (Qiagen). The appropriate amount of first‐strand cDNA template was mixed with specific TaqMan primers (Applied Biosystems, Foster City, CA, USA), RNase‐free water and PCR Master Mix (Applied Biosystems, Foster City). Quantitative RT‐PCR was conducted using a 7900HT Fast Real‐time PCR system (Applied Biosystems, Foster City). All experiments were performed on three independently isolated cDNA samples (*n* = 3 mice). In all cases, expression was compared with that of β‐actin measured from the same sample in parallel on the same plate, giving a threshold (C_t_) difference (ΔC_t_) for β‐actin minus the test gene. Reactions in which the test gene was undetectable were assigned a C_t_ value of 40. The following primer pairs were used (Applied Biosystems, Foster City): *Galr1*: Mm00433515_m1, *Kcnj3*: Mm00434618_m1, and *Kcnj5*: Mm01175829_m1.

### Primary murine intestinal cultures

C57BL6 mice aged 15–21 weeks were culled, and the intestines were collected into ice‐cold Leibovitz (L‐15) medium (Sigma). Mixed intestinal cultures were produced, as previously described (Reimann *et al.*, 2008). Briefly, following the removal of the muscle layers, the intestine was opened longitudinally, rinsed in PBS and cut into 1–2 mm^2^ pieces. Duodenal cultures contained tissue from the top 10 cm of the small intestine distal to the stomach, and ileal cultures contained tissue from the bottom 10 cm of the small intestine, proximal to the caecum. Tissue was digested with 0.3 mg mL^−1^ Collagenase (Sigma), centrifuged at 100× *g* and resuspended in DMEM (24 mM glucose) supplemented with 10% FBS, 2 mM glutamine, 100 U mL^−1^ of penicillin and 0.1 mg mL^−1^ of streptomycin. Intestinal cell/crypt suspensions were plated onto 24‐well plates coated with 1% *v*/*v* Matrigel (BD Bioscience, Oxford, UK) and incubated overnight at 37°C in 5% CO_2_. Each intestinal segment (duodenum, ileum or colon) of an individual mouse gave rise to a single 24‐well plate, and each 24‐well plate only contained tissue isolated from one mouse.

### Secretion studies

Secretion studies on primary intestinal cultures were performed approximately 24 h after plating. Cultures were washed thoroughly and incubated with test reagents in saline solution (see Solutions and chemicals) supplemented with 0.1% fatty acid‐free BSA for 2 h at 37°C. Supernatant and lysate samples were collected. Samples were assessed for GLP‐1 content using a total GLP‐1 assay (Meso Scale Discovery, Gaithersburg, MD, USA). GIP was measured using a total GIP ELISA kit (Millipore, Billerica, MA, USA). GIP and GLP‐1 were measured from the same samples. Hormone secretion was calculated as a percentage of total hormone content per well, to account for an unknown number of L cells per well in the mixed primary cell cultures. Statistical analysis was carried out on the % hormone secretion values. Each graph represents five independent experiments (cultures/24‐well plates).

### cAMP FRET measurements

Single‐cell measurements of cAMP levels were made using intestinal cultures from transgenic mice expressing the FRET‐based sensor *Epac2camps* (Nikolaev *et al.*, 2004) under the control of the proglucagon promoter. Three transgenic mice were used to obtain cells for the FRET experiments. The protocol used was based on the one previously described for GLUTag cells (Moss *et al.*, 2012). Briefly, primary duodenal cells, continuously perifused with saline solution with or without test reagents, were visualised with a ×40 oil immersion objective on an inverted microscope (Olympus IX71, Southend on Sea, UK). Excitation at 435 nm was achieved using a xenon arc lamp coupled to a monochromator (Cairn Research, Faversham, UK) controlled by MetaFluor software (Molecular Devises, Wokingham, UK). CFP emission at 470 nm and YFP emission at 535 nm were monitored using an Optosplit II beam splitter (Cairn Research) and an Orca‐ER digital camera (Hamamatsu, Welwyn Garden City, UK) and expressed as the CFP/YFP fluorescence ratio (Friedlander *et al.*, 2011; Moss *et al.*, 2012). Data were smoothened with a sliding average across 30 s. Maximum ratios were determined at baseline (30 s period prior to test condition) and following test reagent application, and calculated increments were normalised to baseline.

### Materials

The saline solution contained (in mM) 138 NaCl, 4.5 KCl, 4.2 NaHCO_3_, 1.2 NaH_2_PO_4_, 2.6 CaCl_2_, 1.2 MgCl_2_ and 10 HEPES (±10 glucose, as indicated). Saline with 10 mM glucose was used in all secretion experiments for GIP analysis. Unless otherwise stated, all drugs and chemicals were obtained from Sigma UK. Where possible, drugs were made up as a 1000× stock as per manufacturer instructions. Galanin (1–29), the GAL_1_ receptor agonist M617 (Lundstrom *et al.*, 2005) and TPN‐Q (Jin *et al.*, 1999a; Jin & Lu*,* 1999b) were obtained from Tocris Bioscience (Abingdon, UK). The GIRK1/4 (K_ir_3.1/K_ir_3.4) channel activator ML297 was obtained from Abcam Biochemicals (Cambridge, UK).

### Data analysis and statistical procedures

#### Quantitative RT‐PCR

All experiments were performed on three independently isolated cDNA samples (*n* = 3 mice). However, in line with Curtis *et al.* (2015), as *n* < 5, no statistical analysis has been presented. Data are expressed as geometric mean (2^ΔCT) ± SEM.

#### Secretion studies

Hormone secretion data (%GLP‐1, %GIP) were analysed by linear regression using cluster–robust standard error estimation (Huber, 1967), to account for non‐independence of observations made on each culture (mouse). Each graph represents data from five independent experiments/cultures (24‐well plates). Statistical analysis was carried out using the statistical software stata® 14 (College Station, Texas, USA). The threshold for significance was *P* < 0.05. Data are expressed as mean ± SD.

#### Imaging

For CFP/YFP emission ratio data, linear regression using cluster–robust standard error estimation was used to estimate the mean of the paired differences. The threshold for significance was *P* < 0.05. Data are expressed as mean ± SD.

## Results

### *Galr1* mRNA is enriched in enteroendocrine K and L cells

**Figure 1:**
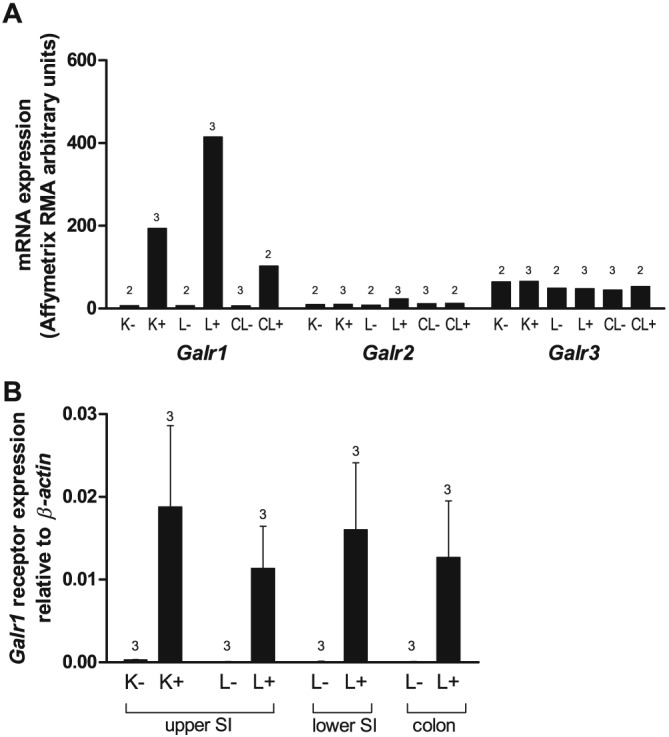
*Galr1* mRNA is enriched in enteroendocrine K and L cells. (A) Mean microarray robust multichip average (RMA) intensities for probes against *Galr1*, *Galr2* and *Galr3* in K cells (K+ cells), small intestinal and colonic L cells (L+ and CL+ respectively) and non‐fluorescent control cells from the same tissue preparations (K‐, L‐ and CL‐ respectively) (*n* = 2–3 each, as indicated). (B) Relative expression of *Galr1* mRNA relative to β‐actin assessed by RT‐PCR in FACS‐sorted cell populations derived from the upper and lower small intestine (SI) and colon. Data are presented as the geometric mean and upper SEM (*n* = 3 mice each).

To identify the galanin receptor expressed by primary incretin‐secreting enteroendocrine cells, FACS‐purified L and K cells from GLU‐Venus and GIP‐Venus mice, respectively, along with non‐fluorescent control cells were analysed by microarray. The only galanin receptor expressed at meaningful levels and enriched in the enteroendocrine populations was the GAL_1_ receptor (Figure 1A). Quantitative qPCR using mRNA from additional FACS‐sorted cells also demonstrated marked enrichment of the GAL_1_ receptor in enteroendocrine L and K cells (Figure 1B).

### Galanin and a GAL_1_ receptor agonist inhibit GLP‐1 and GIP secretion

**Figure 2:**
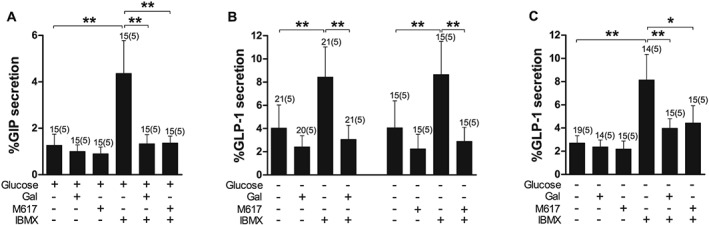
Inhibition of IBMX‐stimulated GIP and GLP‐1 secretion by galanin (Gal) and GAL_1_ receptor agonism. (A) GIP secretion from primary duodenal cultures treated with IBMX (100 μM) with or without Gal (100 nM) and the GAL_1_ receptor agonist M617 (100 nM) in the presence of 10 mM glucose. (B,C) GLP‐1 secretion from primary duodenal (B) and ileal (C) cultures treated with IBMX (100 μM) with or without Gal (100 nM) and the GAL_1_ receptor agonist M617 (100 nM) in the absence of glucose. Data represent mean % hormone secretion ± SD. The number of wells that contributed to the column mean is displayed above each column; the number of independent cultures/experiments/mice is found in brackets. Statistical significance was assessed by linear regression using cluster–robust standard error estimation. *P < 0.05, **P < 0.01.

GIP secretion from K cells in mixed primary small intestinal cultures was significantly stimulated (~3.5‐fold) by incubation with the non‐specific PDE inhibitor IBMX (100 μM) (Figure 2A). IBMX also significantly stimulated GLP‐1 secretion from L cells in duodenal and ileal cultures (~2‐ to 3‐fold, Figure 2B and C). Galanin (100 nM) and the GAL_1_ receptor agonist M617 (100 nM) significantly inhibited IBMX‐stimulated GIP and GLP‐1 secretion from duodenal and ileal cultures (Figure 2). Galanin and M617 also suppressed basal GLP‐1 secretion from the duodenum; however, this effect did not achieve statistical significance (Figure 2B).

### Galanin significantly reduces forskolin‐stimulated intracellular cAMP response in L cells

**Figure 3:**
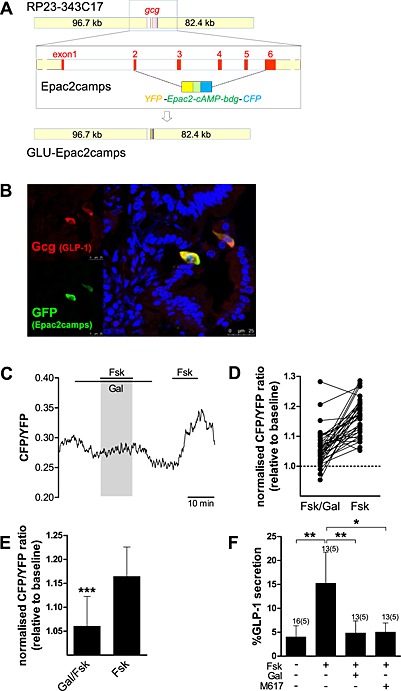
Galanin (Gal) reduces the Fsk‐stimulated cAMP and GLP‐1 responses in primary duodenal L cells of GLU‐*Epac2camps* mice. (A) Schematic diagram of genetic alteration of the GLU‐*Epac2camps* transgene. (B) Correct expression of *Epac2camps* in upper small intestinal L cells of the GLU‐Epac lines was confirmed by immunohistochemistry and confocal microscopy. Representative photomicrograph demonstrating co‐localisation of glucagon (GLP‐1, red fluorescence) and GFP (*Epac2camps*, green fluorescence). Nuclei were visualised with Hoechst staining (blue). (C) Changes in cAMP concentration in response to GAL_1_ receptor/Gα_i_ activation. Primary duodenal L cells were perfused with Fsk (2 μM) with or without Gal (100 nM), as indicated. CFP and YFP emission was monitored in response to excitation with 435 nm. An example trace representing the CFP/YFP ratio of a single cell is depicted. (D) All individual cAMP increases relative to baseline (36 cells) in response to Fsk/Gal and Fsk alone are shown. (E) Mean changes in the CFP/YFP emission ratio in response to application of Fsk with or without Gal in experiments performed as in (C). Data are means ± SD (36 cells, from five cultures/independent experiments/mice). Linear regression using cluster–robust standard error estimation was used to estimate the mean of the paired differences. ***P < 0.001 compared with Fsk alone. (F) GLP‐1 secretion from primary murine duodenal cultures treated with Fsk (2 μM) with or without Gal (100 nM) and the GAL_1_ receptor agonist M617 (100 nM). Data represent mean % hormone secretion ± SD. The number of wells that contributed to the column mean is displayed above each column; the number of independent cultures/experiments/mice is found in brackets. Statistical significance was assessed by linear regression using cluster–robust standard error estimation. *P < 0.05, **P < 0.01.

Forskolin, a potent activator of adenylyl cyclase, stimulates GLP‐1 secretion via an increase in intracellular cAMP ([cAMP]i) in duodenal cultures (Figure 3C–E). L cells within duodenal cultures derived from GLU‐Epac2camps mice were imaged to determine the effect of galanin on the forskolin‐induced [cAMP]i response. In the presence of galanin, the increase in [cAMP]i stimulated by forskolin was significantly inhibited (Figure 3C–E).

### GIRK1/4 (K_ir_3.1/K_ir_3.4) subunits are enriched in enteroendocrine K and L cells

**Figure 4:**
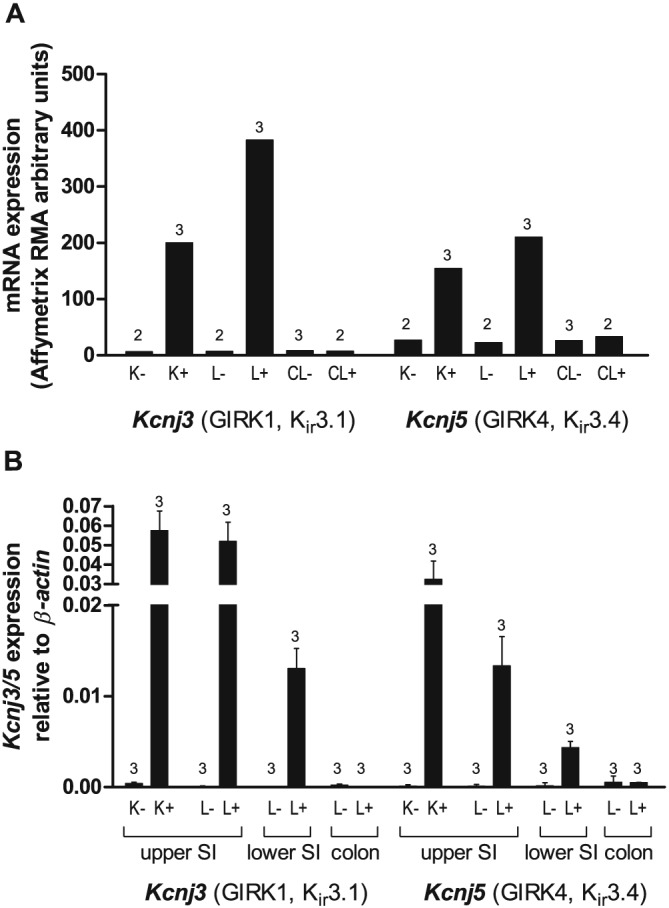
Expression of GIRK channels in enteroendocrine K and L cells. (A) Mean microarray robust multichip average (RMA) intensities for probes against *Kcnj3* (GIRK1, K_ir_3.1) and *Kcnj5* (GIRK4, K_ir_3.4) in K cells (K+ cells), small intestinal and colonic L cells (L+ and CL+ respectively) and non‐fluorescent control cells from the same tissue preparations (K‐, L‐ and CL‐ respectively) (*n* = 2–3 each as indicated). (B) Relative expression of *Kcnj3* and *Kcnj5* mRNA relative to β‐actin assessed by RT‐PCR in FACS‐sorted cell populations derived from the upper and lower small intestine (SI) and colon. Data are presented as the geometric mean and upper SEM (*n* = 3 mice each).

To determine whether GIRK (K_ir_3) channels are expressed by primary incretin‐secreting enteroendocrine cells, FACS‐purified L and K cells from GLU‐Venus and GIP‐Venus mice, respectively, along with non‐fluorescent control cells were analysed by microarray. The only GIRK channel subunits expressed at meaningful levels and enriched in the enteroendocrine populations of the small intestine were GIRK1/4 (K_ir_3.1/K_ir_3.4), which are known to co‐assemble (Chan *et al.*, 1996; Duprat *et al.*, 1995; Krapivinsky *et al.*, 1995; Spauschus *et al.*, 1996) (Figure 4A). Quantitative qPCR using non‐amplified mRNA from additional FACS‐sorted cells also demonstrated marked enrichment of *Kcnj3* and *Kcnj5* in small intestinal enteroendocrine L and K cells (Figure 4).

### GIRK (K_ir_3) activation inhibits basal and stimulated GLP‐1 secretion

**Figure 5:**
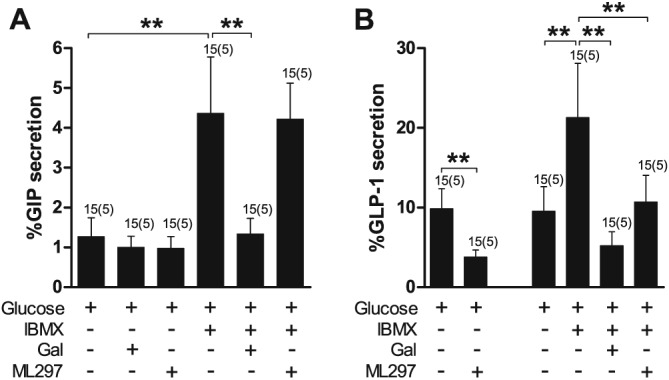
Inhibition of IBMX‐stimulated GLP‐1 but not GIP secretion by ML297, an activator of GIRK channels. (A) GIP secretion from primary duodenal cultures treated with IBMX (100 μM) with or without galanin (Gal) (100 nM) and the GIRK channel agonist ML297 (100 μM) in the presence of 10 mM glucose. (B) GLP‐1 secretion from primary duodenal cultures treated with IBMX (100 μM) with or without Gal (100 nM) and the GIRK channel agonist ML297 (100 μM) in the presence of 10 mM glucose. Data represent mean % hormone secretion ± SD. The number of wells that contributed to the column mean is displayed above each column; the number of independent cultures/experiments/mice is found in brackets. Statistical significance was assessed by linear regression using cluster–robust standard error estimation. **P < 0.01.

GIRK1/4 (K_ir_3.1/K_ir_3.4) channels were activated using ML297, a potent GIRK activator (EC_50_ for GIRK1/4 ≈ 1 μM; Kaufmann *et al.*, 2013). Incubation of duodenal cultures with ML297 (100 μM) significantly inhibited glucose‐stimulated and IBMX‐stimulated GLP‐1 secretion in duodenal cultures (Figure 5B). However, ML297 had no effect on basal or IBMX‐induced GIP secretion (Figure 5A).

### Inhibition of GIRK (K_ir_3) channels or K_ATP_ (K_ir_6) channels does not abolish galanin‐mediated inhibition of GLP‐1

**Figure 6:**
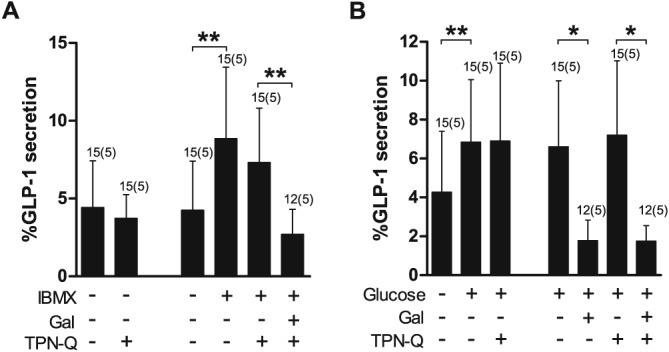
Inhibition of IBMX‐stimulated GLP‐1 secretion by galanin (Gal) is not affected by GIRK channel inhibition. (A) GLP‐1 secretion from primary duodenal cultures treated with IBMX (100 μM) and Gal (100 nM) in the presence or absence of the GIRK channel blocker TPN‐Q (10 μM) and following a 30 min pretreatment with TPN‐Q (10 μM). (B) GLP‐1 secretion from primary duodenal cultures treated with glucose (10 mM) and Gal (100 nM) in the presence or absence of the GIRK channel blocker TPN‐Q (10 μM) and following a 30 min pretreatment with TPN‐Q (10 μM). Data represent mean % hormone secretion ± SD. The number of wells that contributed to the column mean is displayed above each column; the number of independent cultures/experiments/mice is found in brackets. Statistical significance was assessed by linear regression using cluster–robust standard error estimation. *P < 0.05, **P < 0.01.

TPN‐Q is a high‐affinity stable derivative of tertiapin from honey bee venom and selectively inhibits GIRK1/4 and ROMK1 channels with nanomolar affinities (Jin & Lu, 1999b). ROMK1 (*Kcnj1*) is not expressed in enteroendocrine L and K cells according to our microarray data (data not shown). Pretreatment (30 min) and incubation of duodenal cultures with TPN‐Q (10 μM) did not affect the ability of galanin to inhibit IBMX‐stimulated or glucose‐stimulated GLP‐1 secretion (Figure 6). Inhibition of K_ATP_ channels using tolbutamide and gliclazide, at established concentrations (Proks *et al.*, 2013; Reimann *et al.*, 2008), also did not prevent galanin from inhibiting GLP‐1 secretion in response to IBMX (Supporting Information Figure 2).

## Discussion and conclusions

We have demonstrated that galanin potently inhibits both GLP‐1 and GIP secretion from primary small intestinal cultures by acting on the GAL_1_ receptor, which we have identified as the only galanin receptor highly expressed and enriched in enteroendocrine L and K cells. Using a novel transgenic mouse strain, GLU‐*Epac2camps*, which expresses the YFP/CFP‐based cAMP‐FRET‐sensor *Epac2camps* under the control of the proglucagon promoter, we showed that galanin significantly inhibits the intracellular accumulation of cAMP triggered by Fsk in primary L cells. This finding is consistent with the G_i_ coupling of the GAL_1_ receptor. L and K cells were also shown to express GIRK1/4 (K_ir_3.1/K_ir_3.4) channels, which have previously been associated with GAL_1_ receptor signalling. However, we were unable to demonstrate a role for GIRK activation in the inhibition of incretin secretion by galanin.

Enteroendocrine secretion is known to be under the influence of several peptides derived from the enteric nervous system (Psichas *et al.*, 2015) such as members of the bombesin/gastrin‐releasing peptide family and calcitonin gene‐related peptide, which trigger GLP‐1 secretion in various settings (Dumoulin *et al.*, 1995; Herrmann‐Rinke *et al.*, 1995; Plaisancie *et al.*, 1994). However, despite the likely importance of inhibitory influences in fine‐tuning or terminating incretin secretion, they have received relatively little attention. In humans, there is some evidence to suggest that galanin reduces gastrointestinal motility and suppresses the initial postprandial rise in plasma PYY and GLP‐1 but not GIP (Bauer *et al.*, 1989). Furthermore, galanin has been shown to dose‐dependently inhibit GLP‐1 secretion from isolated vascularly perfused rat ileum (Herrmann‐Rinke *et al.*, 1996) and cholecystokinin secretion from STC‐1 cells (Chang *et al.*, 1995) in response to a variety of stimulants including bombesin and Fsk. We demonstrated that galanin is a potent inhibitor of both GLP‐1 and GIP secretion from primary murine small intestinal cultures.

Investigation into the mechanisms underlying the inhibitory effect of galanin on gut hormone secretion has been limited. Chang *et al.* (1995) and Saifia *et al.* (1998) demonstrated that the inhibitory mechanism was sensitive to pertussis toxin in rat ileal cells and STC‐1 cells, respectively, suggesting the recruitment of a G_i_‐coupled pathway. However, the identity of the galanin receptor and the nature of the inhibition, whether direct or indirect, had not been determined. We demonstrate here for the first time that mRNA for the G_i_‐coupled galanin 1 receptor (*Galr1*) is highly enriched in enteroendocrine L and K cells. Moreover, incubation of primary small intestinal cultures with the selective GAL_1_ receptor agonist M617 significantly inhibited GLP‐1 and GIP secretion in the presence of IBMX. In accordance with galanin activating a G_i_‐coupled pathway, the presence of galanin significantly inhibited the increase in intracellular cAMP mediated by Fsk in primary L cells, as measured using cultures from mice expressing the *Epac2camps* sensor under the control of the proglucagon promoter.

Galanin receptor activation has also been associated with the opening of GIRK (K_ir_3.1/K_ir_3.4) channels, which as inward rectifiers play a fundamental role in maintaining the resting membrane potential (Counts *et al.*, 2002; de Weille *et al.*, 1989; Parsons *et al.*, 1998; Smith *et al.*, 1998). Gβγ subunits released from pertussis toxin‐sensitive G_i_ proteins are thought to bind directly to GIRK channels resulting in permeability to K^+^ ions and consequent hyperpolarisation (Huang *et al.*, 1995; Logothetis *et al.*, 1987; Pfaffinger *et al.*, 1985; Reuveny *et al.*, 1994; Wickman *et al.*, 1994). We demonstrate here for the first time that mRNA for the GIRK subunits 1 and 4 (K_ir_3.1/K_ir_3.4) is enriched in enteroendocrine L and K cells. Furthermore, incubation of primary duodenal cultures with the GIRK channel activator ML297 is capable of inhibiting GLP‐1 secretion in the presence of IBMX and/or glucose. Conversely, ML297 did not inhibit GIP secretion. The reasons for this are unclear but might reflect a failure of GIRK mRNA translation in K cells or a higher non‐GIRK resting potassium conductance in K cells compared with L cells. In addition, it is possible that at 100 μM, ML297 is exhibiting off‐target effects on voltage‐gated *ether‐a‐go‐go*‐related gene (*Kcnh2*, Kv11.1) potassium channels (Kaufmann *et al.*, 2013), which are expressed in L cells but not K cells (data not shown). Therefore, we sought to determine whether galanin may activate GIRK1/4 channels as part of its mechanism of action in L cells. However, incubating with the GIRK blocker TPN‐Q did not affect the ability of galanin to inhibit either IBMX‐stimulated or glucose‐stimulated GLP‐1 secretion. TPN‐Q was used at a concentration ~1000‐fold higher than the K_i_ for GIRK1/4. However, the lack of a positive control for TPN‐Q activity in L cells prevents us from conclusively discounting a role for these channels in galanin‐mediated inhibition of GLP‐1 secretion.

K_ATP_ channels (K_ir_6) have also been implicated in the inhibition of gut hormone secretion mediated by galanin (Saifia *et al.*, 1998). We have previously reported that K_ATP_ channel subunits are expressed in primary enteroendocrine L and K cells (Parker *et al.*, 2009; Reimann *et al.*, 2008). However, using the well‐characterised sulfonylureas tolbutamide and gliclazide to block K_ATP_ channels in duodenal cultures, we found no evidence to suggest involvement of these channels in the suppression of GLP‐1 secretion by galanin.

Considerable effort has been made to identify stimulants of incretin secretion. However, our understanding of the physiological regulation of gut hormone secretion is incomplete without knowledge of the contribution of inhibitory neuropeptides and hormones acting simultaneously to prevent, dampen down or terminate secretion. We have used an *in vitro* primary cell model to investigate the inhibitory effect of galanin on gut hormone secretion. As with all *in vitro* models, there are several inherent limitations including the loss of innervation, blood supply and polarity of cells in culture. It is difficult to ascertain the potential impact of these artificial conditions. However, as the inhibitory effects of galanin have previously been demonstrated in both the *ex vivo* and *in vivo* settings (Bauer *et al.*, 1989; Herrmann‐Rinke *et al.*, 1996) with similar results, we are confident that our primary cell culture model is a valid tool and, in conjunction with novel transgenic mouse models, has provided important mechanistic insight.

Previous studies revealed that galanin‐immunoreactive nerve fibres could be readily detected in the vicinity of L cells (Herrmann‐Rinke *et al.*, 1996). However, the general consensus was that a space of several micrometres separated nerve fibres and enteroendocrine cells. Thus, it was argued that enteroendocrine cells were targeted by neuropeptides via diffusion, a method of neurotransmission commonly employed by the enteric nervous system, rather than by synaptic transmission. Recent work (Bohorquez *et al.*, 2014; Bohorquez *et al.*, 2015) now suggests that enteroendocrine cells possess a prominent cytoplasmic process referred to as a “neuropod,” which is capable of direct contact with nerve fibres. Therefore, it is evident that we do not fully understand the interaction between efferent neurotransmission and the fine‐tuning of gut hormone secretion and nutrient responsiveness. Further work is warranted in this emerging field.

In conclusion, we have shown in primary cells that galanin inhibits GLP‐1 and GIP secretion via the GAL_1_ receptor and activation of G_i_‐coupled signalling leading to a reduction in intracellular cAMP. Enteroendocrine L cells also express GIRK1/4 (K_ir_3.1/K_ir_3.4) channels, and the GIRK‐agonist ML297 inhibited GLP‐1 secretion, presumably opening a potassium conductance. However, we found no evidence to suggest that GIRK1/4 or K_ATP_ channel activation plays a role in galanin‐mediated inhibition of GLP‐1 secretion.

## Author contributions

A. P., L. L. G. and F. R. performed the research. A. P., F. R. and F. M. G. designed the research study. A. P. and S. J. S. analysed the data. A. P., F. R. and F. M. G. wrote and edited the paper.

## Conflict of interest

The authors declare that they have no conflicts of interest.

## Supporting information




**Table S1** Oligonucleotides used to create and verify GLU‐Epac2camps mice.
**Table S2** Immunohistochemical characterization of GLU‐Epac2camps mice (small intestine, n=3 mice per strain; colon, n=2 mice per strain).
**Figure S1** Epac2camps in colonic L cells from a Glu‐Epac2camps mouse. Fixed colonic slices were co‐immunostained for GFP (representing Epac2camps) together with glucagon (Gcg, GLP‐1). Nuclei were visualised with Hoechst staining.
**Figure S2** Inhibition of IBMX‐stimulated GLP‐1 secretion by galanin is not affected by K_ATP_ (K_ir_6) channel inhibition. GLP‐1 secretion was measured from primary duodenal cultures treated with IBMX (100μM) and Gal (100nM) in the presence or absence of the K_ATP_ channel blockers Tolbutamide (100μM) and Gliclazide (100nM), in the absence of glucose. Data represent mean % hormone secretion ± SD. The number of wells that contributed to the column mean is displayed above each column; the number of independent cultures/experiments/mice is found in brackets. Statistical significance was assessed by linear regression using cluster‐robust standard error estimation. *P<0.05, **P<0.01.

Supporting info item
