# Supplementary material for: Galanin inhibits GLP‐1 and GIP secretion via the GAL1 receptor in enteroendocrine L and K cells
Source: Br J Pharmacol. 2016 Feb 8;173(5):888–98. doi: 10.1111/bph.13407 (PMC4761093; doi:10.1111/bph.13407)
Supplement: Supplementary file 1 — Supporting info item [file BPH-173-888-s001.pdf]

Supplementary Table 1: Oligonucleotides used to create and verify GLU-Epac2camps mice

Name	Sequence
Epac_in1	GGG GGA AGA AGG TAC CTC CTG
Epac_in2	CAC GGA TCC TTT CAG AAT GAT G
Epac_in3	TCT AGA CTC AAC GTC CCT CAG
mGLP002	TAC ATC CCA AGT GAC TGG CAC GAG ATG TTG TGA AGA TGG TTG TGA ATG GTG AAA TAC CTA CTT GTA CAG CTC GTC CAT GCC GAG
mGLP005	CAT CTG CAT GCA AAG CAA TAT AGC
mGLP006	TGC TCC CCC ATC ACC CCC TAC CCA CCC CCA TTC TGT GTT CCA TCA GGC AGA AAA AAA ATC CAC CAT GGT GAG CAA GGG CGA G
mGLP007	TGC TCC CCC ATC ACC CCC
mGLP008	TAC ATC CCA AGT GAC TGG CAC
mGLU008	AAT TGA GCT CAT TTG GAC TGC C
mGLP009	ATA AAG CTT GCT CCC CCA TCA CC
mGLP010	ATA CTC GAG TAC ATC CCA AGT GAC TGG
mGLP011	ATA CTC GAG TCT AAT GCT GCC TGA TG
mGLP012	TGA ATT TAA CAT GGC ATT GGA GC
mGLP013	ATC CAG GTG TTG TGA CTG CG
FRGLU008	AAT TGA GCT CAT TTG GAC TGC C
RM41	AAG GTA GAG TGA TGA AAG TTG TT
RM42	CAC CAT GTC CTC TGT CTA TTC

Supplementary Table 2: Immunohistochemical characterization of GLU-Epac2camps mice (small intestine, n=3 mice per strain; colon, n=2 mice per strain)

Glu-Epac Mice strain	Strain 20 Small intestine	Strain 21 Small intestine	Strain 20 Colon	Strain 21 Colon
Number of GFP+ve cells investigated	250	585	184	195
Number of GFP+ve cells that were glucagon+ve	233	481	177	186
**% GFP+ve cells that were glucagon+ve**	**93.2%**	**82.2%**	**96.2%**	**95.4%**
Number of glucagon+ve cells Investigated	311	556	205	213
Number of glucagon+ve cells that were GFP+ve	233	481	177	186
**% glucagon+ve cells that were GFP+ve**	**74.9%**	**86.5%**	**86.3%**	**87.3%**

Immunohistochemistry and Immunofluorescence Microscopy

Tissues from GLU-Epac2camps mice, strains 20 and 21, were fixed in 4% paraformaldehyde, dehydrated in 15% and 30% sucrose and frozen in optimal cutting temperature embedding media (VWR, Lutterworth, UK). Cryostat-cut sections (7-10 µm) were mounted directly onto polylysine-covered glass slides (VWR, Leuven, Belgium). Slides were incubated for 1 hour in blocking solution containing 5% donkey serum/1% BSA and overnight in PBS/0.05% Triton-X/1% BSA/5% serum with primary antisera of interest: GFP/YFP (ab5450, abcam, Cambridge, UK, 1:1000) and glucagon (Sc-13091, Santa Cruz Biotechnology, Dallas, Texas, USA, 1:200). Sections were washed with blocking solution and incubated with appropriate secondary antisera (donkey AlexaFluors 488 and 546, Life Technologies, Paisley, UK) diluted 1:300. Control sections were stained with secondary antisera alone. Sections were washed with PBS and mounted with Prolong Gold (Life Technologies, Loughborough, UK) before confocal microscopy (Leica TCS SP8 X, Wetzlar, Germany).

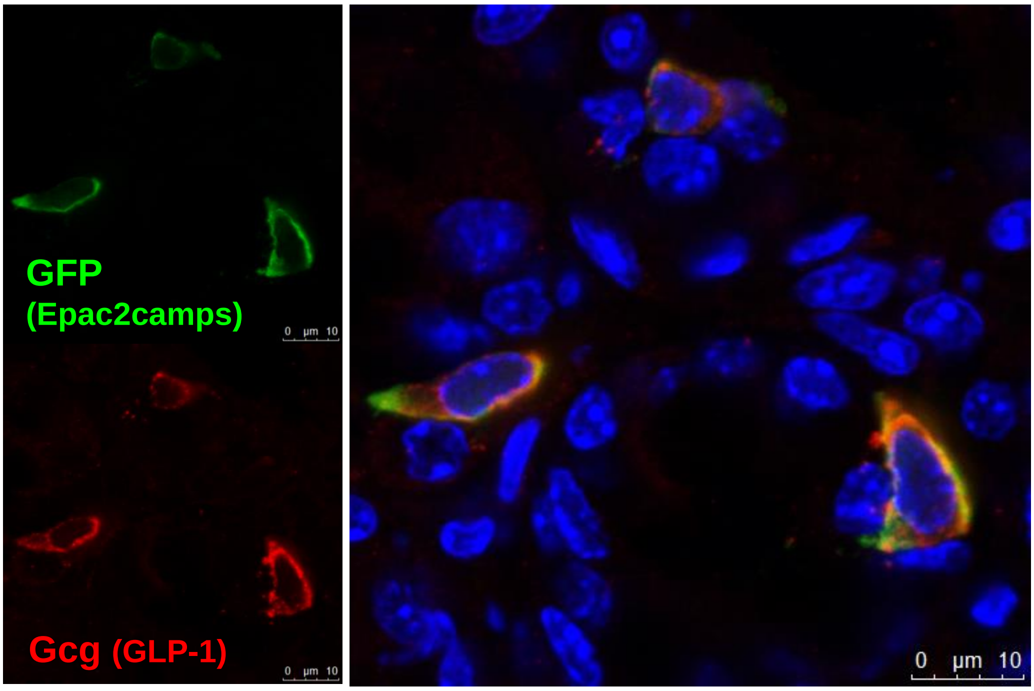

Supplementary Figure 1. Epac2camps in colonic L cells from a Glu-Epac2camps mouse. Fixed colonic slices were co-immunostained for GFP (representing Epac2camps) together with glucagon (Gcg, GLP-1). Nuclei were visualised with Hoechst staining.

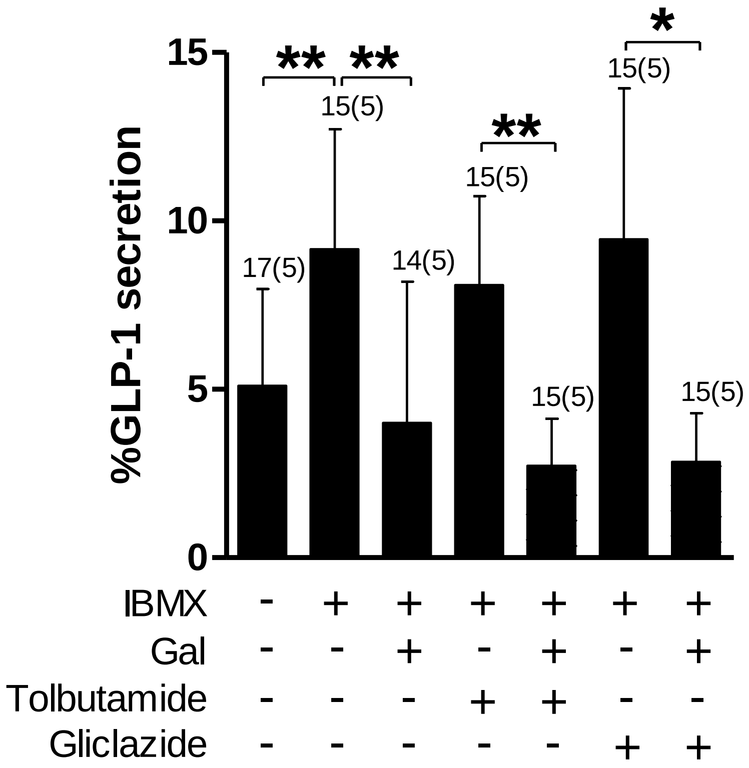

Supplementary Figure 2. Inhibition of IBMX-stimulated GLP-1 secretion by galanin is not affected by K_{ATP} ($K_{ir}6$) channel inhibition. GLP-1 secretion was measured from primary duodenal cultures treated with IBMX (100µM) and Gal (100nM) in the presence or absence of the K_{ATP} channel blockers Tolbutamide (100µM) and Gliclazide (100nM), in the absence of glucose. Data represent mean % hormone secretion ± SD. The number of wells that contributed to the column mean is displayed above each column; the number of independent cultures/experiments/mice is found in brackets. Statistical significance was assessed by linear regression using cluster-robust standard error estimation. *P<0.05, **P<0.01
